# 98. Multispecies Outbreak of *Klebsiella pneumoniae* Carbapenemase-3 (KPC-3)-producing Enterobacterales in an Acute Care Hospital – Virginia, 2023-2024

**DOI:** 10.1093/ofid/ofae631.035

**Published:** 2025-01-29

**Authors:** Steven D Langerman, Danielle A Rankin, Clarissa Bonnefond, Mohammed Khan, Amara Fazal, Marissa Grossman, Shaina Bernard, Richard A Stanton, Paige Gable, Heather Moulton-Meissner, Judith Noble-Wang, Alison L Halpin, Matthew Arduino, Erin Breaker, Thomas Ewing, Thao Masters, Anthony Harrington, Kristen Clancy, Lauren Turner, Alexandra Lorentz, Katelin Gali, Stephanie Neal, Emily Hawker, Kayleigh Rehkopf, Maroya S Walters, Patricia Bair

**Affiliations:** Centers for Disease Control and Prevention, Atlanta, GA; Centers for Disease Control and Prevention, Atlanta, GA; Virginia Department of Health, Winchester, Virginia; Centers for Disease Control and Prevention, Atlanta, GA; Centers for Disease Control and Prevention, Atlanta, GA; Centers for Disease Control and Prevention, Atlanta, GA; Virginia Department of Health, Winchester, Virginia; Division of Healthcare Quality Promotion, Centers for Disease Control and Prevention, Atlanta, GA; Centers for Disease Control and Prevention, Atlanta, GA; Centers for Disease Control and Prevention, Atlanta, GA; Centers for Disease Control and Prevention, Atlanta, GA; Centers for Disease Control and Prevention, Atlanta, GA; Centers for Disease Control and Prevention, Atlanta, GA; Centers for Disease Control and Prevention, Atlanta, GA; Centers for Disease Control and Prevention, Atlanta, GA; Centers for Disease Control and Prevention, Atlanta, GA; Centers for Disease Control and Prevention, Atlanta, GA; Centers for Disease Control and Prevention, Atlanta, GA; Virginia Department of General Services | Division of Consolidated Laboratory Services, Richmon, Virginia; Virginia Department of General Services | Division of Consolidated Laboratory Services, Richmon, Virginia; Virginia Department of General Services | Division of Consolidated Laboratory Services, Richmon, Virginia; Virginia Department of Health, Winchester, Virginia; Virginia Department of Health, Winchester, Virginia; Virginia Department of Health, Winchester, Virginia; Centers for Disease Control and Prevention, Atlanta, GA; Virginia Department of Health, Winchester, Virginia

## Abstract

**Background:**

Plasmid-mediated outbreaks have been associated with gaps in infection control (IC) and environmental reservoirs such as sink drains. In October 2023, the Virginia Department of Health (VDH) identified a cluster of four *Klebsiella pneumoniae* carbapenemase (KPC)-producing *Klebsiella variicola* urinary tract infections among patients at Acute Care Hospital A (ACH A). VDH and CDC collaborated to investigate the source and prevent further transmission.

**Figure:**
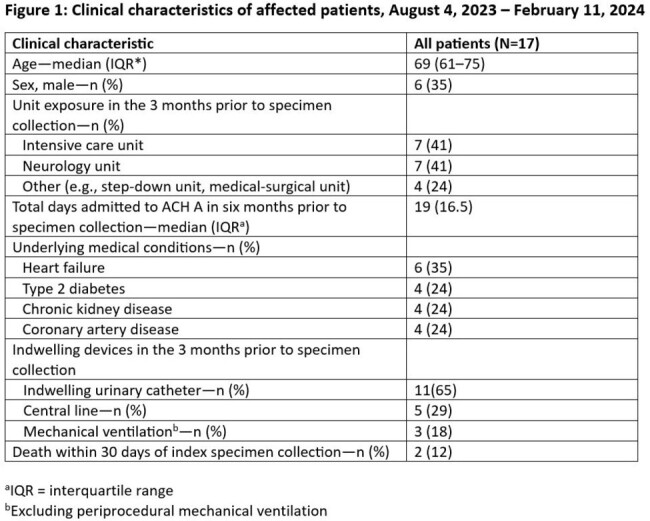

**Methods:**

We reviewed medical records, directly observed IC practices (e.g., hand hygiene, sink hygiene, environmental cleaning), performed active surveillance in units with cases, and collected environmental samples. Clinical and environmental isolates harboring KPC underwent short-read whole-genome sequencing (WGS); long-read WGS was performed on a subset of isolates. We defined a case as *K. variicola* or other Enterobacterales with an IncN plasmid marker and *bla*_KPC-3_, *bla*_TEM-1_, *dfrA14*, and *qnrS* antimicrobial resistance genes from any specimen source in an ACH A patient.

**Figure:**
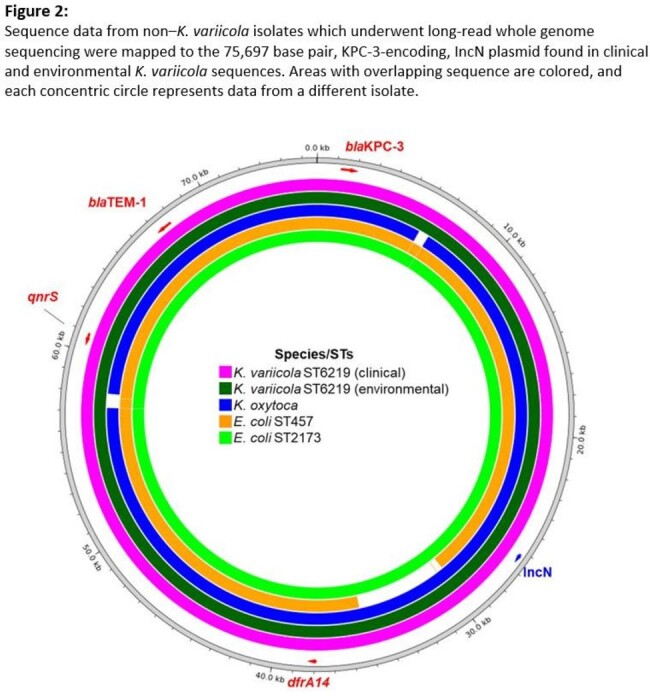

**Results:**

From August 4, 2023 to February 11, 2024, we identified 18 cases from 17 patients (urine: 11, rectal swab: 6, tracheal aspirate: 1 [Figure 1]). Organisms identified included *K. variicola* (14), *Escherichia coli* (2), *K. oxytoca* (1), and *K. aerogenes* (1). We observed gaps in hand hygiene adherence, disposal of antibiotics in handwashing sinks, storage of patient care supplies in sink splash zones, and gaps in environmental cleaning. KPC-producing *K. variicola* was recovered from a sink drain and an aerator in two sinks in one patient room. Clinical and environmental *K. variicola* isolates varied from 0-10 single nucleotide variants over 93.5% of a 5.3 Mbp core genome. The *E. coli*, *K. oxytoca*, and *K. aerogenes* cases had sequence overlaps of 92.5-100% with the IncN plasmid isolated from a clinical *K. variicola* (Figure 2).

**Conclusion:**

Detection of KPC in an uncommon organism, *K. variicola*, led to the discovery of an outbreak of multiple Enterobacterales strains harboring a common plasmid. Clonal expansion and plasmid-mediated spread across species can occur in parallel in patients and environmental reservoirs. Facilities experiencing an increase in detection of a carbapenemase across multiple organisms should consider the potential for a plasmid outbreak.

**Disclosures:**

**All Authors**: No reported disclosures

